# Supplementary material for: Developmental coordination disorder: “It’s not on people’s radars… They’re not interested”
Source: PLoS One. 2025 Nov 18;20(11):e0337001. doi: 10.1371/journal.pone.0337001 (PMC12626263; doi:10.1371/journal.pone.0337001)
Supplement: S1 File — (DOCX) [file pone.0337001.s001.docx]

**Agenda for parental interviews**

1. Can you tell us about the process you went through to get your child DCD/ Dyspraxia diagnosis?
*Prompt(s):*

*Did you encounter any barriers?

Was there any helpful facilitators?

Age of diagnosis?*

*2.* Did you feel sufficiently supported by the NHS and healthcare professionals during and after the diagnostic process?

*Prompt(s):*

*Did you come up against any scepticism or doubt? (If yes, can you tell me about this?)*

*Were you confronted by any non-believers? (If yes, how did you cope with this?)*

*Did you have confidence in the professionals managing your child’s diagnosis (can you tell
me why? Were they experts?)*

3. Did you feel sufficiently supported by your family (immediate and wider) after your child’s diagnosis?

*Prompt(s):*

*Did you come up against any scepticism or doubt? (If yes, can you tell me about this?)*

*Were you confronted by any non-believers? (If yes, how did you cope with this?)*

*Did you feel supported at home? (Can you describe why?)*

4. Did you reach out to your friends after your child’s diagnosis? *(can you tell me why?)*

*Prompt(s):*

*Beyond friends and family did you reach out for support elsewhere (e.g. support groups) –
can you tell me why?*

*Did you come up against any scepticism or doubt? (If yes, can you tell me about this?)*

*Were you confronted by any non-believers? (If yes, how did you cope with this?)*

5. Do you think your child’s diagnosis has impacted on their experience in school in relation to peer and teacher perceptions and support?

*Prompts:
Did you feel sufficiently supported by your school when telling them about your child’s diagnosis?
Were there any positive impacts?
Were there any negative impacts?*

6. Do you think your child’s diagnosis has influenced their friendships?

*Prompts:
Have their friends been supportive?
Do they share their diagnosis/ difficulties with friends?
Do you think their diagnosis has ever been problematic in terms of friendships?*

7. Do you feel like your child is understood in relation to their diagnosis?
