# Supplementary material for: Developmental coordination disorder: “It’s not on people’s radars… They’re not interested”
Source: PLoS One. 2025 Nov 18;20(11):e0337001. doi: 10.1371/journal.pone.0337001 (PMC12626263; doi:10.1371/journal.pone.0337001)
Supplement: Attachment — Submitted filename: Response to reviewers .docx [file pone.0337001.s005.docx]

**Reviewer 1**

Thank you for addressing the reviewer's comments.

There is just one minor revision I would request. Currently, there are two sub-themes called 'Healthcare'. I would change one of these so they are distinctive.

We thank the reviewer for highlighting duplicated subtheme names. We have now amended these as follows:

Inadequate knowledge and awareness within Healthcare

Inadequate support within Healthcare

**Reviewer 2**

The revision shows a number of improvements, sufficient consideration of reviewer concerns, and the new table helps improve the readability.

We thank the reviewer for their kind words.
